# Ostéome ostéoide intra-articulaire du genou simulant un tableau d'arthrite du genou

**DOI:** 10.11604/pamj.2015.22.337.8435

**Published:** 2015-12-04

**Authors:** Mohamed Amine Karabila, Tarik Madani, Mohamed Azouz, Younes Mhamdi, Ismail Hmouri, Mohamed Kharmaz, Ahmed Bardouni, Abdou Lahlou, Mustapha Mahfoud, Mohamed Saleh Berrada

**Affiliations:** 1 Service de Chirurgie Orthopédique et de Traumatologie, CHU Ibn Sina, Rabat, Maroc

**Keywords:** Ostéome, genou, arthrite, Osteoma, knee, arthritis

## Abstract

L'ostéome ostéoïde est une tumeur bénigne ostéoblastique relativement rare, constituée d'une petite lésion de tissu charnu et vascularisé, appelé nidus, cernée d'une ostéocondensation réactionnelle. Il représente 2 à 3% de l'ensemble des tumeurs osseuses et 10 à 20% des tumeurs osseuses bénignes et survient généralement entre 10 et 25 ans (extrêmes: 5-53 ans), avec une nette prédominance masculine. La localisation intra-articulaire est rare représentant environ 10 à 13% descas. La clinique et les images radiologiques, souvent atypiques, rendent son diagnostic ardu. Le risque de traitement inadéquat est important. Jusqu’à 40% d'arthroscopies inappropriées ont été rapportés. Le cas rapporté illustre cette situation.

## Introduction

L'ostéome ostéoïde est une tumeur osseuse bénigne qui affecte les adultes jeunes et se localise préférentiellement au niveau des os longs. La symptomatologie clinique est alors atypique et peut faire errer le diagnostic constituant un défi diagnostique pour les cliniciens Nous rapportons une observation d'ostéome ostéoïdeintra-articulaire du genou révélé par des douleurs de type inflammatoire évoluant dans un contexte rapide et nous essayons de décrire les particularités clinico-radiologiques et les modalités thérapeutiques de cette localisation.

## Patient et observation

**Figure 1:**
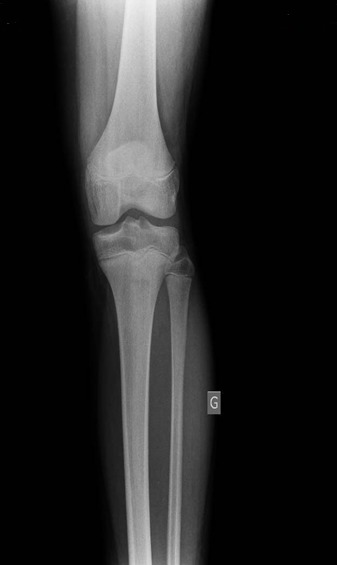
Radiographie de face du genou gauche

**Figure 2:**
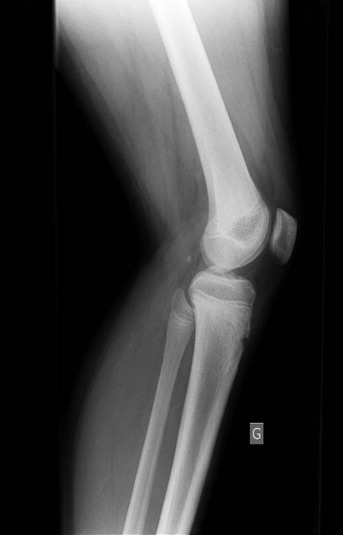
Radiographie de profil du genou gauche

**Figure 3:**
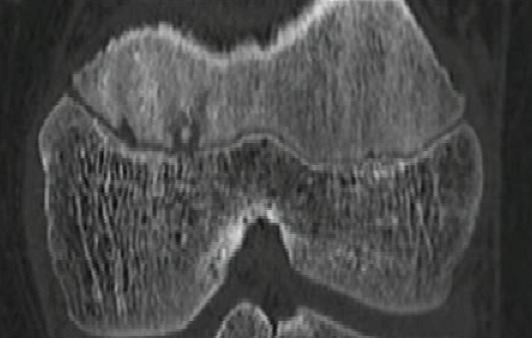
Incidence coronale du scanner de genou gauche montrant le nidus au contact du cartilage de croissance

**Figure 4:**
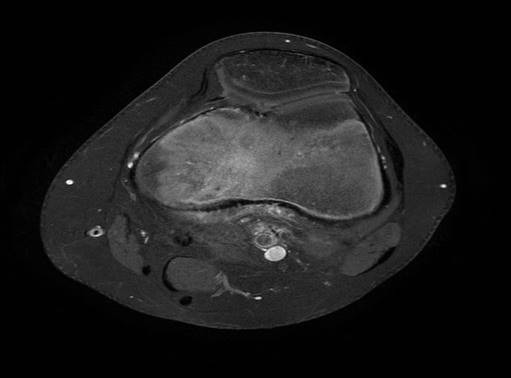
IRM du genou montrant l’œdème péri-lésionnel du condyle interne

**Figure 5:**
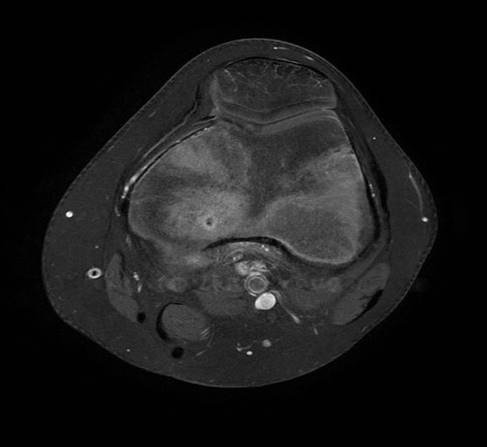
IRM du genou montrant le nidus

**Figure 6:**
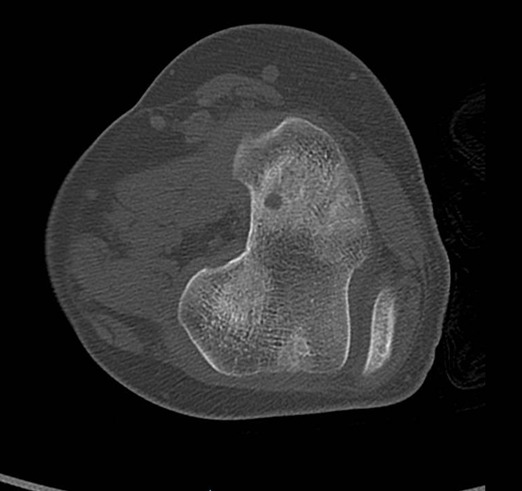
Image scanographique au moment du repérage de la lésion pour forag

En mars 2014, un enfant de 14 ans, sans antécédents notables, vient se présenter aux urgences pédiatriques pour une douleur aigue de genou gauche. La douleur était ressentie au sport depuis 2 jours au niveau de la face interne du genou en regard de l'interligne sans notion de traumatisme évident avec l'apparition d'un pic fébrile à 39° 24 heures après la symptomatologie. Elle augmentait progressivement en intensité et devenait peu à peu constante. L'examen clinique trouve un ‘dème du genou avec limitation importante et douloureuse des mouvements du genou gauche associée à une fièvre à 38°. La biologie a montré un syndrome inflammatoire modéré, une NFS normale, la vitesse de sédimentation à la première heure à 17mm et une CRP à 28.5 mg/l. Les radiographies du genou ont été interprétées comme normales (Figure 1, Figure 2). Alors vu ces données une arthrite du genou a été suspectée et une ponction du genou était pratiquée avec un résultat non significatif. Un lavage articulaire du genou était fait sous arthroscopie avec la réalisation des prélèvements à titre bactériologique qui sont avérés négatifs. L’évolution après 2 mois s’était marquée par la persistance de cette douleur avec une boiterie à la marche. Une IRM du genou était demandée à la recherche d'une lésion méniscale ou une ostéochondrite ou une lésion tumorale et qui a montré un œdème du compartiment postéro-externe du genou avec une image nodulaire de 10 mm de diamètre (Figure 3, Figure 4). Une tomodensitométrie du genou a complété cet examen et a confirmé la présence d'une lésion hypodense nodulaire à centre hyperdense à ras du cartilage de croissance du condyle interne (Figure 5) faisant évoquer le diagnosticd'ostéomeostéoide. L'exploration est complétée par une scintigraphie osseuse qui montre une hyperfixation intense au niveau de la lésion puis le patient a été adressé au service de radiologie où il a bénéficié d'une résection par forage sous repérage scannographique (Figure 6). L'examen anatomopathologique d'exérèse a confirmé alors le diagnostic. La symptomatologie douloureuse a complètement disparu en quelques jours à la suite de l'intervention. Le patient a repris rapidement ses activités sportives, sans restriction et à 12 mois de l'intervention, l'enfant présente un genou sec et indolore sans signe de récidive.

## Discussion

L'ostéome ostéoïdeintra-articulaire est rare (10-13% des cas). Il touche majoritairement la hanche et représente un défi diagnostic [1–3]. Un ostéome ostéoideintra-articulaire (OOIA) évolue fréquemment dans un contexte trompeur [4] retardant le diagnostic et la prise en charge adéquate [5]. Les symptômes cliniques les plus communs décrits au cours des OOIA sont des douleurs articulaires, des synovites, une raideur ou une tuméfaction des parties molles et une diminution des mobilités articulaires [6]. L'examen clinique est souvent peu spécifique. Des pathologies mécaniques ou microtraumatiques sont généralement évoquées: lésions méniscales, bursopathies, tendinopathies, chondromalacie, corps étrangers [4]. La présentation clinique est atypique. Szendroi et al. [2] ont comparé les délais diagnostiques entre les ostéomes ostéoïdesintra-articulaires et les autres localisations. Le délai moyen était de 26,6 mois pour les ostéomes intra-articulaires, contre 8,5 mois pour les autres localisations. Les particularités radiologiques des ostéomes intraarticulaires sont autant de pièges. L'image classique de nidus, bordée d'une ostéosclérose périphérique est le plus souvent absente (50-75%). La radiologie conventionnelle est soit normale, soit caractérisée par une ostéopénie périarticulaire locale [1, 2]. Toutes ces particularités cliniques et radiologiques des OO intra-articulaires imposent le recours fréquent à plusieurs moyens d'imagerie [2]. La scintigraphie osseuse, qui garde une place parmi les différents moyens diagnostics avec une sensibilité atteignant les 100% [7], révèle une, fixation localisée « en spot » précoce et intense. Elle ne permet pas de confirmer le diagnostic positif, mais son intérêt réside de rechercher d'autres localisations généralement exceptionnelles et sert à cibler précisément le reste du bilan d'imagerie (TDM et IRM). Depuis les années 1990, les techniques mini-invasives en percutanée après repérage du nidus par broche sous contrôle tomodensitométrique [8] représentent le traitement de choix. Avec des équipes entraînées, le taux de succès est de 90-98%. Lors d'une localisation intra-articulaire, il est particulièrement important d'empêcher toute intervention délabrante ou fragilisante, Il faut aussi éviter une approche trans-articulaire, pour prévenir le risque infectieux, minimiser la réaction synoviale et la destruction du cartilage. Le traitement d'une localisation intra-articulaire peut toutefois induire des lésions dégénératives, dont le patient doit être averti, particulièrement dans les petites articulations.

## Conclusion

L'ostéome ostéoïdeintra-articulaire du genou est une lésion difficile à diagnostiquer. La présentation clinique est le plus souvent atypique. Les erreurs d'appréciations sont fréquentes, qui font le lit à de nombreuses procédures thérapeutiques inappropriées. La confrontation de la clinique et de plusieurs moyens d'imagerie est souvent nécessaire. Le traitement doit éviter de provoquer des dégâts cartilagineux. La chirurgie percutanée est la technique de référence pour traiter ces lésions.
